# Lower limit of iron quantification using dual‐energy CT — a phantom study

**DOI:** 10.1002/acm2.13124

**Published:** 2020-12-23

**Authors:** Xia Jiang, David E. Hintenlang, Richard D. White

**Affiliations:** ^1^ Department of Radiology Ohio State University College of Medicine Columbus OH USA

**Keywords:** dual‐energy CT, iron overload, lower limit of quantification, three‐material‐decomposition

## Abstract

**Purpose:**

Dual‐energy computed tomography (DECT) has been proposed for quantification of hepatic iron concentration (IC). However, the lower limit of quantification (LLOQ) has not been established, limiting the clinical adoption of this technology. In this study, we aim to (a) establish the LLOQ using phantoms and (b) investigate the effects of patient size, dose level, energy combination, and reconstruction method.

**Methods:**

Three phantom sizes and eight vials of ferric nitrate solution with IC ranging from 0 to 10 mg/ml were used. DECT scans were performed at 80/140 and 100/140Sn kVp, and using five different levels of CT dose index (CTDI). An image‐domain three‐material‐decomposition algorithm was used to calculate the IC. The LLOQ was determined based on the coefficient of variation from repeated measurements.

**Results:**

The measured IC correlated strongly with the true IC in the small and medium phantoms (R^2^ of linear regression > 0.99) and moderately in the large phantom (0.8 < R^2^<0.9). The LLOQ improved with increased CTDI. At 30 mGy, the LLOQ was found to be 0.50/1.73/6.25 mg/ml in the small/medium/large phantoms, respectively. 80/140Sn kVp resulted in superior LLOQ for all phantom sizes compared to 100/140Sn kVp, primarily due to the difference in their iron enhancement ratios (1.94 and 1.55, respectively). Iterative reconstruction was found to further improve the LLOQ (by ~ 11%), whereas reconstruction kernel smoothness had negligible effect. The LLOQ of iron was significantly higher than that of iodine due to its lack of a useful k‐edge and lower enhancement ratio.

**Conclusion:**

Iron quantification at clinically important levels was achieved in a small‐ and a medium‐sized phantom using DECT, but proved challenging in a large phantom. Wide spectral separation and accurate calibration were found to be critical to the success of the technology.

**Abbreviations**
CTDI_vol_: volumetric computed tomography dose index
DECT: dual‐energy computed tomography
DSCT: dual‐source computed tomography
FBP: filtered back projection
IC: iron concentration
LLOQ: lower limit of quantification
LTS: liquid tissue surrogate
MRI: Magnetic Resonance Imaging
SD: standard deviation
R^2^: coefficient of determination
RMSE: root‐mean‐square error
ROI: region‐of‐interest
SAFIRE: Sinogram Affirmed Iterative Reconstruction
VIC: virtual iron content
VOI: volume‐of‐interest

## 1 INTRODUCTION

Iron overload can have significant clinical consequences. It may result from hereditary hemochromatosis or transfusional hemosiderosis.^1^ The former affects approximately 1 in 300 Caucasians;^2^ the latter occurs in patients with hematological disorders (e.g., thalassemia), who require repetitive blood transfusions. The liver is the primary site of iron storage in the human body, and the hepatic iron concentration (IC) is considered to be a reliable marker for the total body iron load.^3^, ^4^ While percutaneous liver biopsy remains the gold standard for the quantification of the hepatic IC, there is an increasing clinical need for alternative noninvasive methods, especially for blood‐transfusion patients, who are likely to require regular monitoring with risks related to interventions.^5^, ^6^ Due to its ease of measurement, serum or plasma ferritin level, is the most commonly used surrogate for body iron load, although the correlation between the two quantities has been found to be poor in individual patients.^5^ Magnetic Resonance Imaging (MRI) can provide a three‐dimensional mapping of the hepatic IC, based on a shortening of the proton transverse relaxation times T_2_ or T_2_* as a consequence of the paramagnetism of ferritin and hemosiderin.^7^ However, there is currently only one regulatory‐approved MRI technique available in the United States for hepatic iron quantification (FerriScan, Resonance Health, Claremont, WA, Australia), with its widespread use limited by high cost and long scan time.^8^ In addition, the performance of T_2_‐ and T_2_*‐based methods is known to deteriorate at high IC.^7^, ^9^

More recently, dual‐energy computed tomography (DECT) has been proposed for noninvasive liver iron quantification. Phantom^10^, ^11^ and human studies^12^, ^13^, ^14^, ^15^ have shown promising results. However, the lower limit of quantification (LLOQ) has not been established, without which the clinical value of this technology cannot be determined. In addition, it remains to be understood how various technical and clinical parameters could affect the quantification results, including the patient size, radiation dose level, energy combination, and reconstruction methods. In this study, we investigate these issues using phantoms, and identify some of the key factors critical to the reliability and efficacy of the DECT approach to iron quantification.

## 2 MATERIALS AND METHODS

### 2.1 Phantom construction

Three phantoms with square cross‐sections were constructed. The phantoms had water equivalent diameters of 21.3, 30.7, and 40.1 cm, representing the abdominal dimensions of a small, a medium, and a large patient, respectively.^16^ The phantoms were constructed with 9.5‐mm thick acrylic walls and were filled with water. Each phantom contained eight vials of ferric nitrate solutions with IC of 0, 0.1, 0.2, 0.5, 1.0, 2.0, 5.0, and 10.0 mg of iron per ml. The solutions were prepared by adding ferric nitrate nonahydrate to a liver‐equivalent liquid tissue surrogate (LTS) made from six chemicals, which has been shown to exhibit nearly identical x‐ray attenuation properties to that of human tissues over a wide range of photon energy.^17^ All chemicals used had purity ≥99.4% according to the suppliers. A 2‐liter solution was made for each IC to reduce uncertainty. Ferric nitrate nonahydrate was weighed using an analytical balance with linearity better than 0.002 g, while the other chemicals were weighed with an analytical balance with linearity better than 0.05 g. The mass of water component in the nanohydrate was subtracted from the necessary mass of distilled water when preparing the LTS. The volume of the LTS solution was calculated using a physical density of 1.06 g/ml.^17^ The uncertainty in the IC of the finished solutions is estimated to be <3% for the lowest IC and better for other ICs.

**Fig. 1:**
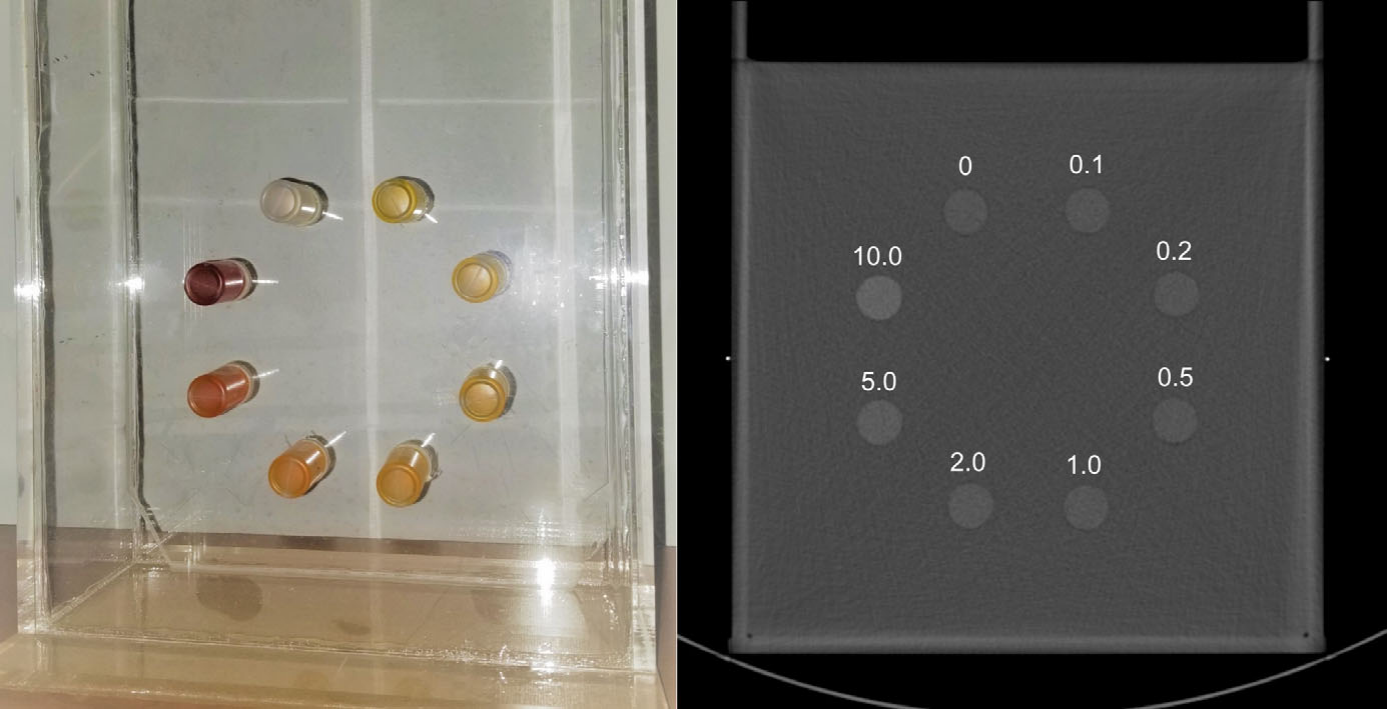
A photographic image (left) and a cross‐sectional CT image (right) of the medium‐sized phantom with eight vials of ferric nitrate solution. The number above each vial indicates the IC in mg/ml.

A photographic and a cross‐sectional CT image of the medium‐sized phantom are shown in Fig. 1. The eight vials were situated in symmetric locations with respect to the diagonal lines of the phantom, so that they were subject to the same amount of beam‐hardening effect from the phantom. The vials were at distances of 6.4, 7.4, and 8.4 cm from the centers of the small, medium, and large phantoms.

### 2.2 DECT acquisition

DECT scans were performed using a second‐generation dual‐source CT (DSCT) (Somatom Definition Flash, Siemens Healthcare, Forchheim, Germany) using the factory‐default abdominal helical DECT protocol (helical pitch = 0.6, rotation time = 0.5 s, collimation = 32 × 0.6 mm, slice thickness/increment = 5/5 mm). The scans were performed with energy combinations of 80/140Sn kVp and 100/140Sn kVp, where “Sn” indicates the use of a tin filter for additional hardening of the high‐energy beam, which improves spectral separation. To test the effect of radiation dose, all phantoms were scanned at five different levels of volumetric CT dose index (CTDI_vol_, body phantom): 5, 10, 15, 20, and 30 mGy (a rotation time of 1.0 s was used for the 30 mGy acquisition to meet the demand of tube output). All scans were repeated five times.

Image reconstruction was performed using filtered back projection (FBP) with dual‐energy specific reconstruction kernels at three different smoothness levels: D10, D30, and D50. In addition, to test the effect of iterative reconstruction, the images were reconstructed using Sinogram Affirmed Iterative Reconstruction (SAFIRE) at strength levels of 3 and 5 with a medium smooth kernel (Q30).

### 2.3 Material decomposition

**Fig. 2:**
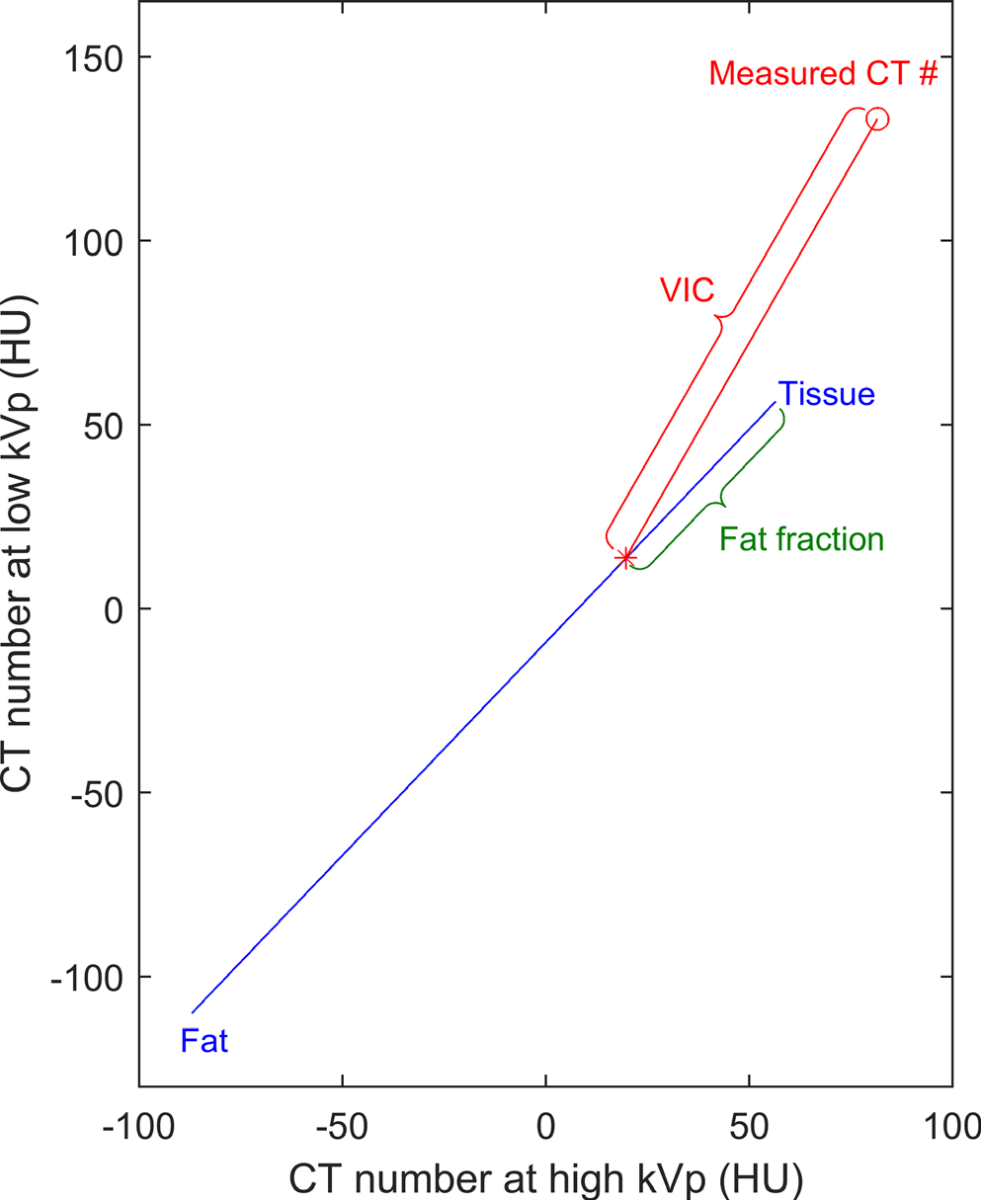
Illustration of the three‐material‐decomposition algorithm. The measured CT numbers at the low and high energies is represented by the red circle, from which a straight line is extended at a slope that is equal to the iron enhancement ratio, until it intersects (at the red asterisk) with the blue line that represents a tissue‐fat mixture. The distance between the circle and asterisk determines the virtual iron content (in HU) and is considered to be proportional to the true iron concentration. The location of the intersection can be used to calculate the fat fraction.

The IC for each vial was calculated using a standard image‐domain three‐material‐decomposition algorithm,^18^ which was implemented using an in‐house script in Matlab (The MathWorks, Inc., Natick, MA) and is illustrated in Fig. 2. Briefly, a straight line is extended from the measured CT numbers at a predetermined slope (termed the iron enhancement ratio), until it intersects with a line that represents a tissue‐fat mixture. The length of the resultant segment is termed the virtual iron content (VIC). The VIC (in HU) is considered to be proportional to the IC (in mg/ml), and is converted to the latter using a fixed conversion factor.

### 2.4 Calibration

To carry out the algorithm described above, several parameters have to be first determined, including the CT numbers for pure tissue, the iron enhancement ratio, and the conversion factor from VIC to IC. The necessary calibration was performed for each phantom size and for each kVp combination. A 1.4‐cm diameter circular region‐of‐interest (ROI) was used to measure CT number in HU and the result was averaged across four adjacent slices. The data from the 20‐mGy and 30‐mGy acquisitions were pooled for the purpose of calibration.

**Fig. 3:**
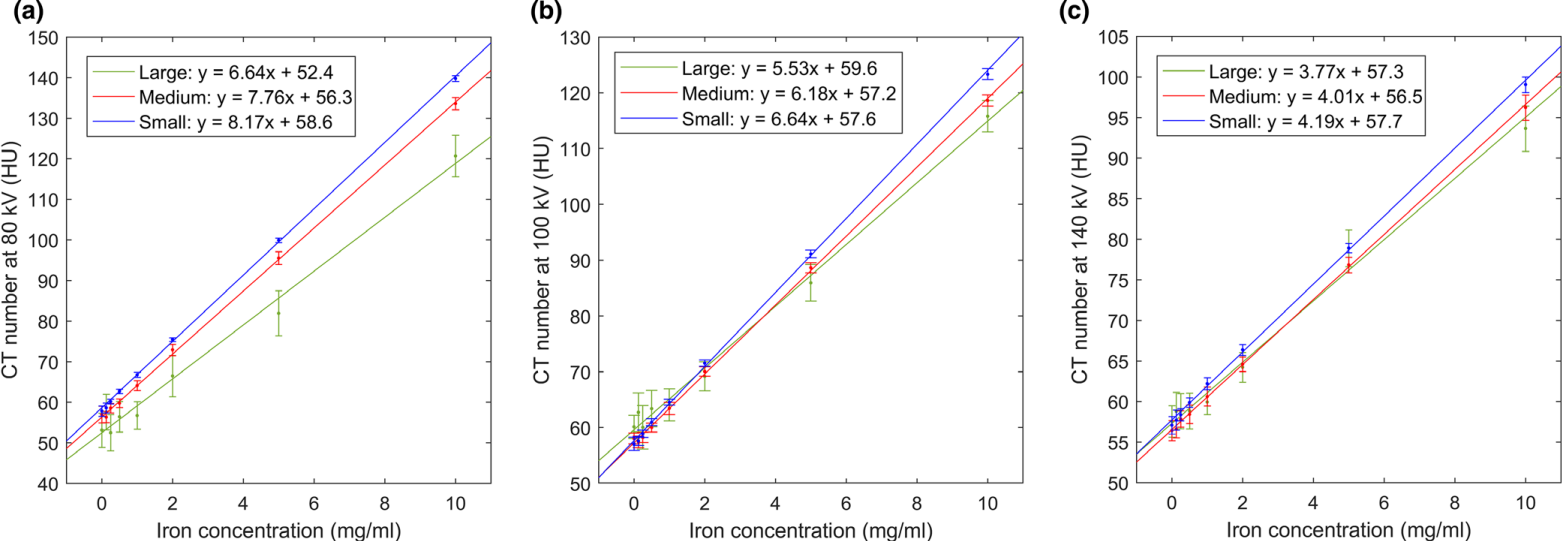
The measured CT numbers at 80 kVp (a), 100 kVp (b), and 140Sn kVp (c) for the small (blue), medium (red), and large (green) phantoms. The straight lines represent the linear regression, whose equations are provided in the figure legends, where “x” and “y” correspond to the IC and the CT number, respectively.

The CT numbers for tissue were determined by performing a least‐square linear regression (LSLR) on the measured CT number as a function of the IC (Fig. 3). The intercepts determined from the LSLR were then used as the CT numbers for tissue. Because the phantoms contained no fat component, no calibration was performed for fat, and fixed CT numbers were used instead (−110, −103, and −87 HU for 80, 100, and 140 Sn kVp, respectively).

**Fig. 4:**
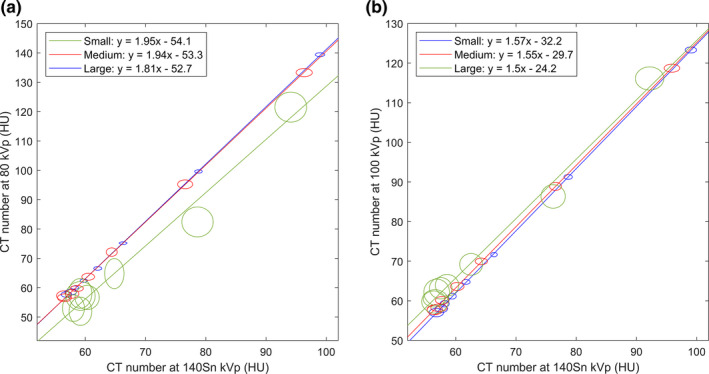
Determination of the iron enhancement ratios for the 80/140Sn kVp (A) and 100/140Sn kVp (B) energy combinations in the small (blue), medium (red), and large (green) phantoms. The measured CT number at each IC is shown as an ellipse, of which the horizontal and vertical radii correspond to the SD at high and low kVp. The straight lines represent the LSLR, whose equations are provided in the figure legends, where “x” and “y” correspond to the CT numbers at the high and low kVp, respectively.

The iron enhancement ratio was determined by performing a two‐variable LSLR^19^, ^20^ on the measured CT numbers at eight ICs (Fig. 4). The slopes obtained from the LSLR were then used as the iron enhancement ratios.

**Fig. 5:**
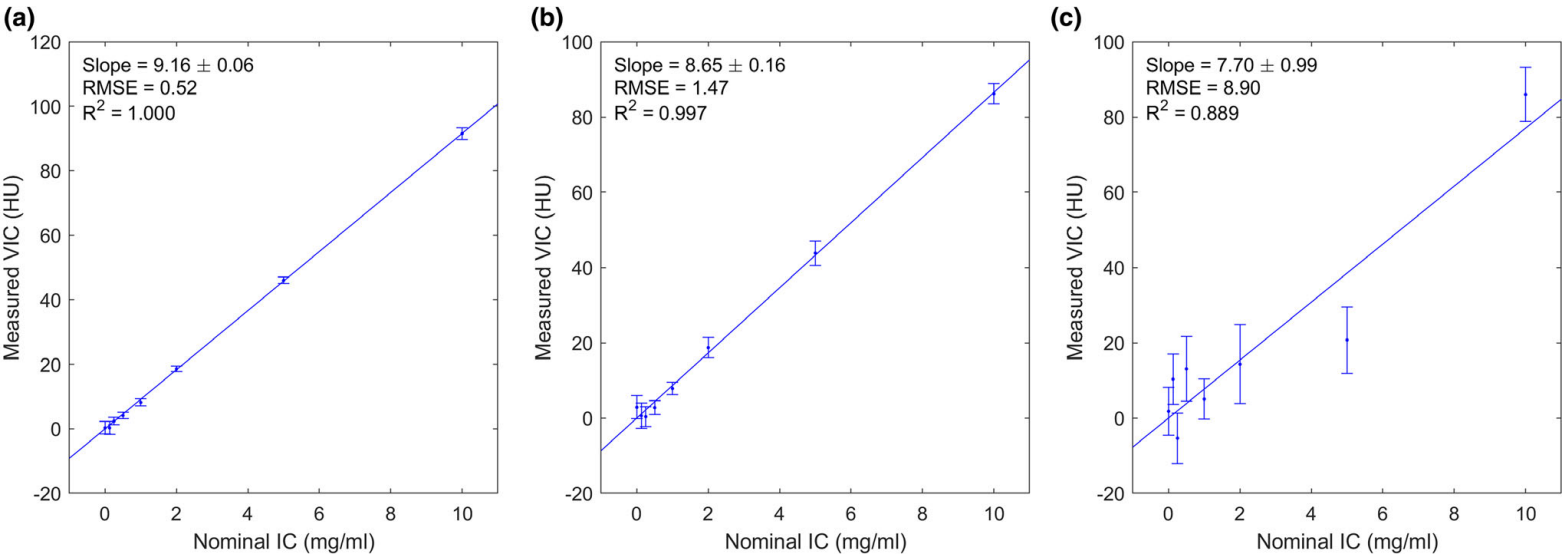
Correlation between the measured VIC and the nominal IC in the small (a), medium (b), and large (c) phantoms. The straight lines are LSLR, whose slopes correspond to the conversion factor and is given in the figure legends. The RMSE and R^2^ of the regression are provided in the legends.

The VIC could then be calculated using the three‐material‐decomposition algorithm illustrated in Fig. 2, and the result was compared to the known ICs in order to obtain the conversion factor (Fig. 5). A LSLR was performed on the calculated VIC as a function of the known IC. The intercept was forced to be zero as the VIC is expected to be proportional to the IC. The conversion factor was calculated as the inverse of the fitted slope. The coefficient of determination (R^2^) and residual root‐mean‐square error (RMSE) of the LSLR were calculated to quantify the goodness of fit.

### 2.5 Determination of LLOQ

The LLOQ was previously defined for DECT‐based iodine quantification by Jacobsen et al.^21^ as the lowest iodine concentration at which the coefficient of variation was ≤20%. We adopt a similar approach and define the LLOQ for iron quantification as the lowest iron concentration at which

(1) $$\frac{\text{max}\left(SD,RMSE\right)}{\mathrm{IC}}\;\leq \;20\%,$$

where SD represents the root‐mean‐square (RMS) of the standard deviations at all eight ICs, RMSE is the residual RMSE between the measured data and the predetermined calibration curve, and the IC is the predicted iron concentration. The rationale for the inclusion of a second term, RMSE, is to more fully account for the potential deviation between a random measurement and the calibration curve (see Discussion).

### 2.6 Additional analysis

To test the effect of using different phantoms for calibration and measurement, we applied the calibration data obtained from the medium phantom to the small and the large phantoms for measurement, and assessed the resultant bias. To assess the impact of CT number inaccuracy, we examined the effect of artificially shifting the CT number in the low‐ or high‐energy image by 3 HU before carrying out the three‐material‐decomposition algorithm. To assess the effect of volume‐averaging on LLOQ, we repeated the procedure describe in the previous section by using only one CT slice, and by averaging the measured VIC over two, three, or four CT slices before determining the LLOQ.

## 3 RESULTS

### 3.1 CT Number

The measured CT numbers at the eight ICs for the three phantom sizes at 80, 100, and 140Sn kVp are shown in Fig. 3. The CT number exhibited a size dependence, especially for lower kVp and higher IC, which could potentially be attributed to a beam‐hardening effect. In all cases, the relationship between the CT number and IC could be adequately described by a linear function, and the parameters of the linear regression are given in the legends of Fig. 3.

### 3.2 Iron enhancement ratio

Figure 4 shows the LSLR used for the determination of the iron enhancement ratio. At 80/140Sn kVp (Fig. 4A), even though the measured CT numbers in the small and medium phantoms were different, they conformed to the same straight line, and therefore resulted in very similar iron enhancement ratios (1.95 and 1.94, respectively). The enhancement ratio in the large phantom was lower at 1.81. Similarly, at 100/140Sn kVp (Fig. 4B), the measured enhancement ratios were closely matched in the small and medium phantoms (1.57 and 1.55, respectively), and lower in the large phantom (1.50). At both kVp combinations, the data from the small and medium phantoms showed excellent linearity, whereas those from the large phantom showed greater deviation from a linear trend.

### 3.3 Conversion factor

The relationship between the calculated VIC and the true IC is shown in Fig. 5, which can be adequately described by a linear fit with an intercept of zero, confirming the proportionality between the two quantities. The slopes of the LSLR are given in the legends of Fig. 5, and the conversion factors from VIC to IC are provided in Table 1. The goodness of fit is also provided in Fig. 5 legend. In the small and the medium phantoms, excellent linearity was observed (R^2^ > 0.99). Whereas in the large phantom, the linearity was weaker (R^2^ = 0.89).

**Table 1:** Estimated conversion factors from VIC (HU) to IC (mg/ml) for different energy combinations and phantom sizes

Energy combination	Small	Medium	Large
80/140Sn kVp	0.109 ± 0.001	0.116 ± 0.002	0.130 ± 0.017
100/140Sn kVp	0.126 ± 0.001	0.132 ± 0.003	0.118 ± 0.018

### 3.4 LLOQ

The estimated LLOQ using the FBP reconstruction is provided in Table 2. As is expected, the LLOQ was found to improve with higher radiation dose. At the same dose level, significant deterioration in the LLOQ was found with increasing phantom dimension. In nearly all cases, the 80/140 Sn kVp energy combination led to superior LLOQ compared to 100/140 Sn kVp.

**Table 2:** LLOQ of IC in mg/ml using the FBP reconstruction

Phantom size and energy combination	CTDI_vol_ (body)				
	5 mGy	10 mGy	15 mGy	20 mGy	30 mGy
Small, 80/140Sn kVp	2.22	**1.07**	**1.06**	**0.99**	**0.50**
Small, 100/140Sn kVp	**2.01**	**1.63**	**2.10**	**1.38**	**1.18**
Medium, 80/140Sn kVp	4.01	2.14	**1.83**	**1.33**	**1.73**
Medium, 100/140Sn kVp	4.95	2.91	**2.46**	**2.33**	**2.55**
Large, 80/140Sn kVp	A	A	*7.77*	*6.10*	*6.25*
Large, 100/140Sn kVp	A	A	*6.88*	*6.89*	*6.85*

Note

**Bold font**: R^2^ > 0.99; normal font: 0.90 < R^2^ < 0.99; *italic font*: R^2^ < 0.90. “A” indicates artifacts present.

The estimated LLOQ using the SAFIRE reconstruction (level 5) is provided in Table 3. For almost all dose levels, energy combinations, and phantom sizes, SAFIRE resulted in improved LLOQ compared to FBP. The average reductions in the LLOQ at SAFIRE strength levels 3 and 5 were ~6.6% and ~11%, respectively. Reconstruction kernel smoothness was found to have negligible effect on the LLOQ.

**Table 3:** LLOQ of IC in mg/ml using the SAFIRE reconstruction at strength level 5

Phantom size and energy combination	CTDI_vol_ (body)				
	5 mGy	10 mGy	15 mGy	20 mGy	30 mGy
Small, 80/140Sn kVp	1.99	**0.85**	**1.01**	**0.93**	**0.46**
Small, 100/140Sn kVp	**1.79**	**1.51**	**1.93**	**1.26**	**1.09**
Medium, 80/140Sn kVp	3.51	1.82	**1.58**	**1.10**	**1.60**
Medium, 100/140Sn kVp	4.42	2.58	**2.18**	**1.93**	**2.27**
Large, 80/140Sn kVp	A	A	*6.64*	*5.54*	*5.90*
Large, 100/140Sn kVp	A	A	*6.32*	*6.76*	*6.85*

Note

**Bold font**: R^2^ > 0.99; normal font: 0.90 < R^2^ < 0.99; *italic font*: R^2^ < 0.90. “A” indicates artifacts present.

In the small and medium phantoms, excellent (R^2^ > 0.99) or strong (0.90 < R^2 ^< 0.99) agreement was observed between the measurements and the calibration curve, and the estimated LLOQ in these cases is considered to be more reliable. Weaker agreement (R^2^ < 0.9) was observed in the large phantom, for which the estimated LLOQ is likely to be less reliable. In the large phantom and at CTDI_vol_ <= 10 mGy, streaking artifacts were present and the images were judged to be not suitable for quantitative analysis.

### 3.5 Additional analysis

**Fig. 6:**
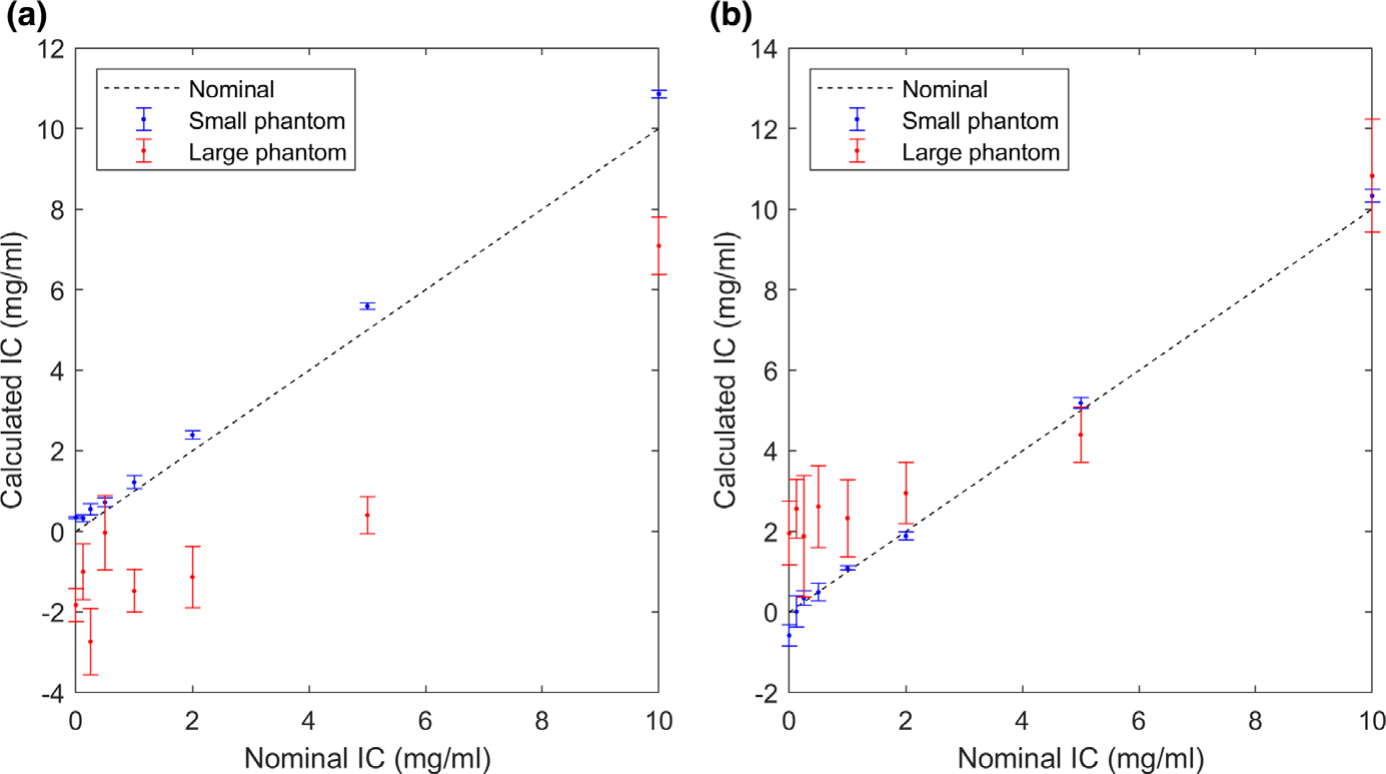
Simulated iron quantification results by applying the calibraton data from the medium phantom to the small (blue) and large (red) phantoms for 80/140Sn kVp (a) and 100/140Sn kVp (b). The broken line represents the true IC.

**Fig. 7:**
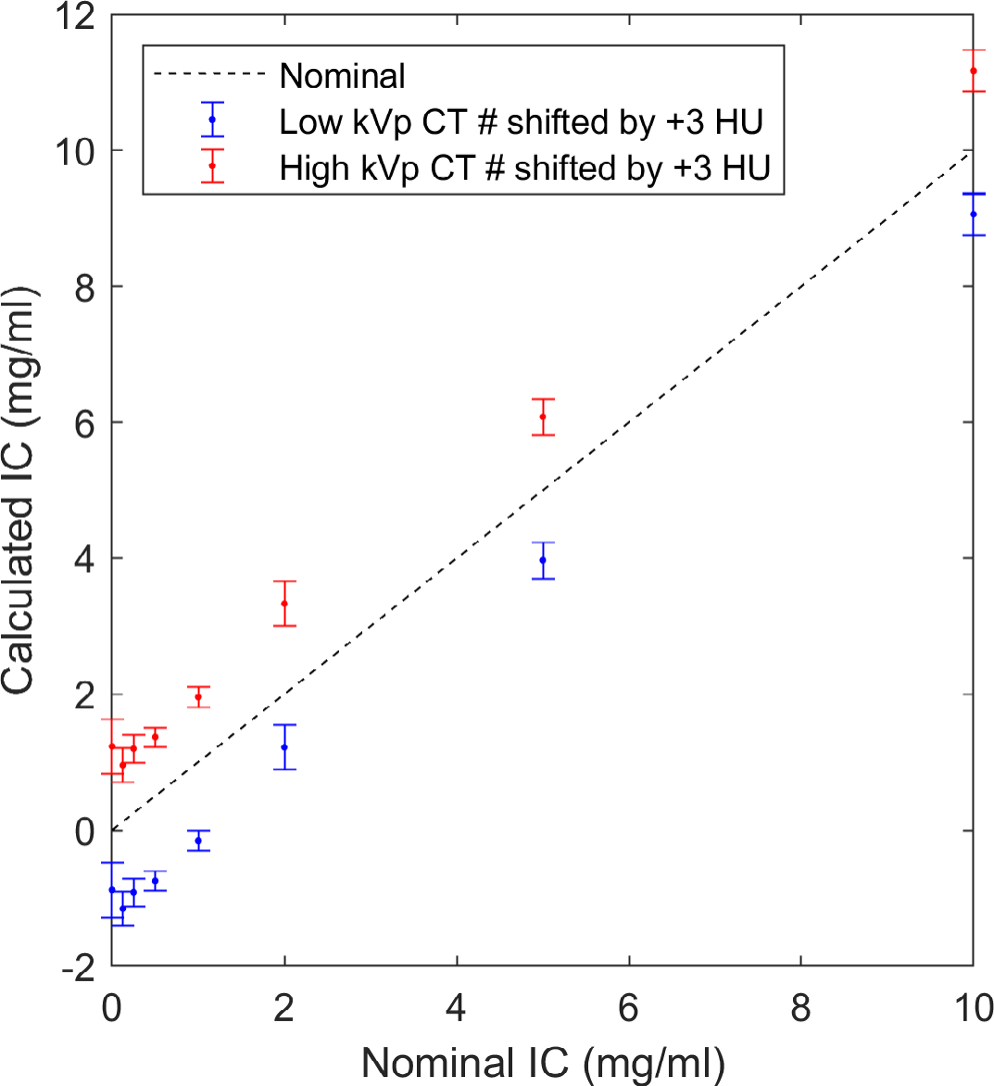
Simulated iron quantification results by aritifically shifting the CT numbers in the low‐ (blue) or high‐ (red) energy image by +3 HU. Results are shown for the medium phantom at 80/140Sn kVp.

**Fig. 8:**
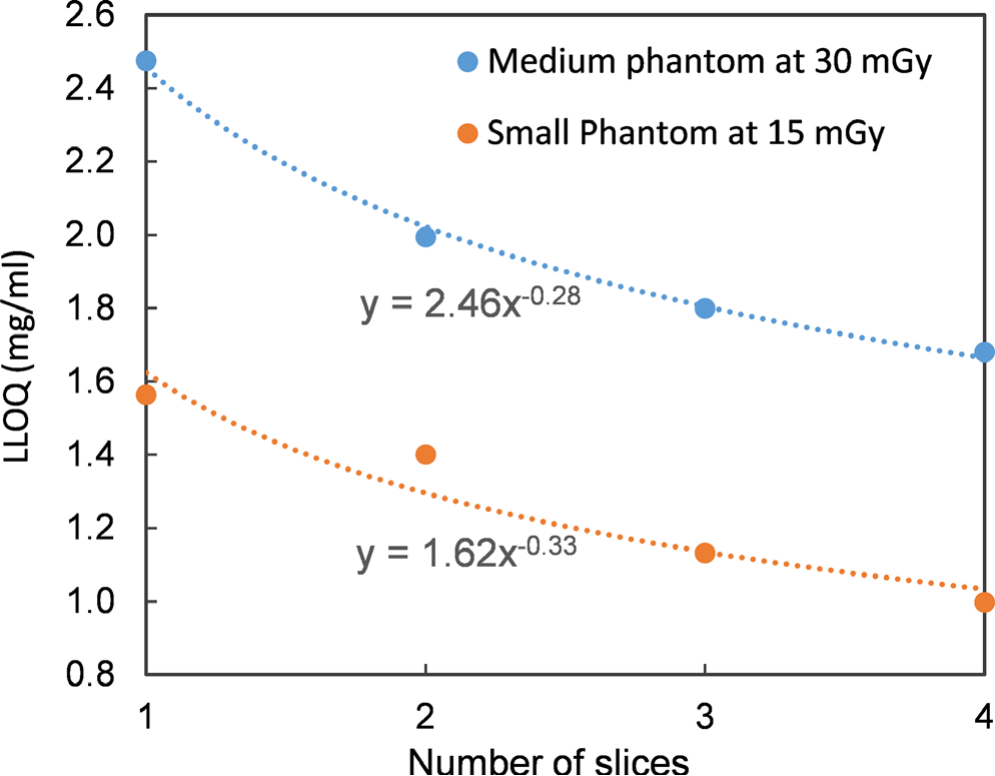
The improvement in LLOQ through volume averaging. The LLOQ was determined by using only one CT slice, and by averaging the calculated VIC over two, three, or four slices. Results are shown for the medium phantom using CTDI_vol_ = 30 mGy (blue) and for the small phantom using CTDI_vol_ = 15 mGy (amber). The equations next to the curves describe the least‐sqaure fit to a power function, where “x” and “y” correspond to the number of slices and the LLOQ.

The bias due to applying the calibration data for the medium phantom to the other two phantoms is reported in Fig. 6 for both energy combinations. The effect of shifted CT number is demonstrated in Fig. 7 for the medium phantom at 80/140 Sn kVp. The effect of volume averaging is illustrated in Fig. 8.

## 4 DISCUSSION

Iron overload is typically managed with iron chelation therapy, with the goal being to maintain hepatic IC in an optimal range of 18 to 38 µmol/g (or 1.1 to 2.2 mg/ml). Hepatic IC greater than 80 µmol/g (or 4.7 mg/ml) is associated with greatly increased risks of cardiac disease and early death.^5^ Our results suggest that quantification of IC at these levels is potentially achievable using DECT. In the small phantom, all three levels can be reliably quantified with CTDI_vol_ >=10 mGy. In the medium phantom, it is difficult to quantify at 1.1 mg/ml, but the other two levels could be reliably quantified with CTDI_vol_ >= 15 mGy. In the large phantom, it would be difficult to achieve reliable quantification at all three levels. However, it should be pointed out that in patients with severe iron overload, the IC can significantly exceed 4.7 mg/ml,^5^, ^8^ in which case DECT may still be useful, even for large patients. As this group of patients likely require more aggressive chelation therapy and more frequent monitoring of the iron load, a fast noninvasive imaging technique would be particularly valuable.

Compared to T_2_‐ and T_2_*‐based MRI techniques for iron mapping, DECT can be completed at faster speed and lower cost. T_2_ relaxometry is known to be less precise with severe iron overload due to the slower change in T_2_ at high IC.^7^ T_2_* relaxometry is similarly compromised at high IC due to rapid signal decay, and is limited to IC below ~30 mg/g dry weight (or ~9.5 mg/ml).^9^ In contrast, DECT potentially becomes increasingly more reliable at higher IC, and therefore may complement MRI for severe iron overload cases. MRI‐based techniques are further confounded by issues such as B_0_ inhomogeneity and the presence of both ferritin and hemosiderin in the liver, which are known to cause different signal relaxations.^7^ DECT is unaffected by these issues. Similar to MRI, DECT can also provide simultaneous fat and iron quantification.^10^

The LLOQ established in this study is significantly higher than the LLOQ previously reported for iodine quantification (0.07–1.0 mg of I/ml).^21^ This difference can be primarily attributed to two reasons: First, iron causes less enhancement in CT number than iodine does. For example, the enhancement due to 10 mg/ml of iron in the medium phantom at 80 kVp was ~78 HU (Fig. 3A). Using the identical phantom with iodine inserts, the enhancement due to 10 mg/ml of iodine was previously measured to be ~375 HU^22^ — a difference by a factor of ~4.8. This difference is due to iron’s lack of a useful k‐edge in the diagnostic energy range (Fig. 923). Second, the iron enhancement ratio (1.94 and 1.55 for 80/140Sn kVp and 100/140Sn kVp, respectively) is also much lower than the iodine enhancement ratio (3.01 and 2.24 for those same energy combinations^22^). The success of the three‐material‐decomposition algorithm requires for the enhancement curve (Fig. 2, red line) to have a sufficiently higher slope than that of the tissue‐fat mixture (Fig. 2, blue line; slope ≈ 1.16). A low enhancement ratio results in inefficient quantification. This is also the cause of the inferior performance of 100/140Sn kVp (Tables 2 and 3), which has an enhancement ratio too close to the slope of tissue‐fat mixture. For this reason, 100/140Sn kVp is likely limited for iron quantification, even for large patients. Consequently, maximizing spectral separation should be a major concern for DECT‐based iron quantification. In this regard, DSCT is likely to outperform other DECT implementations that are currently available, due to its ability to offer wide energy separation by employing two x‐ray tubes. The third‐generation DSCT (Siemens Healthcare, Forchheim, Germany) promises wider spectral separation than the second‐generation DSCT used in the present study, by offering 70/150Sn kVp combination and a thicker Sn filter, and therefore is likely to lead to lower LLOQ than our present results and should be investigated by further studies. The iron enhancement ratios measured in the present study for the small and medium phantoms agree well with a previously reported value (1.9).^10^

**Fig. 9:**
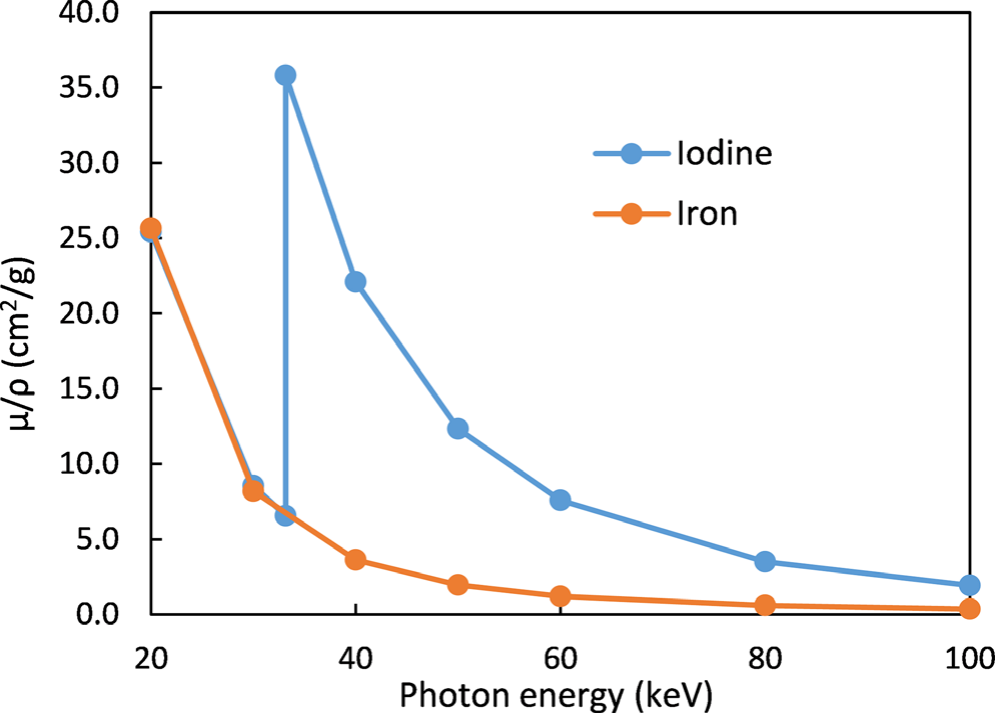
Mass attenuation coefficients (μ/ρ) of iodine (blue) and iron (amber). The two elements have similar µ/ρ below 33 keV. Above 33 keV, iodine contrast benefits significantly from its k‐edge absorption. Iron’s k‐edge (7.1 keV), on the other hand, has negligible impact in the diagnostic energy range. Data from NIST Standard Reference Database 126.^23^

**Fig. 10:**
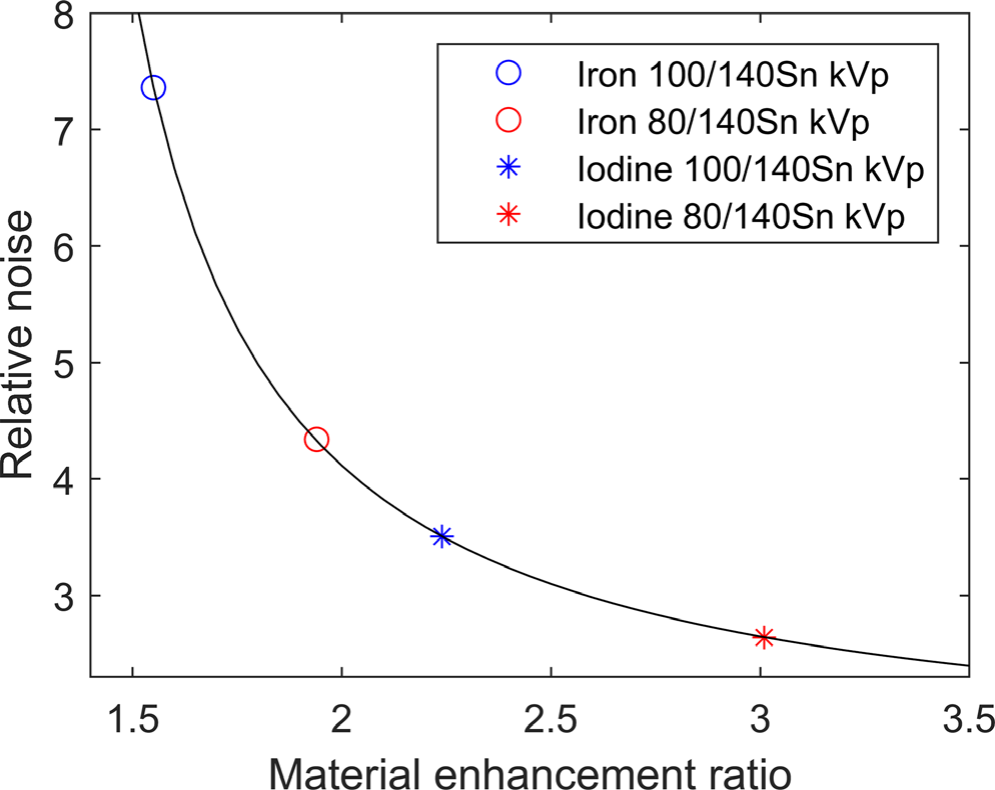
Relative noise in the calculated material content (iron or iodine) image as a function of the material enhancement ratio. It is assumed that the radiation dose is optimally distributed between the low‐ and high‐energy acquisitions, so that the two images have the same amount of Gaussian noise. The relative noise is calculated as the ratio of the noise in the virtual iron or iodine content image to the noise in the low‐ and high‐energy images.

The three‐material‐decomposition algorithm illustrated in Fig. 2 assumes that the presence of iron content does not change the volume of the LTS solution, that is, ferric nitrate causes additional attenuation on top of tissue and fat, without displacing the latter. This is considered justified as the very low IC investigated in this study is expected to cause negligible volume expansion of the solution. The nitrate component of the compound may cause a slight shift in the attenuation along the tissue‐fat dimension (blue line in Fig. 2), but is unlikely to affect the estimation of iron content. The primary source of uncertainty in image‐based three‐material decomposition is image noise. It is known that noise in the calculated material content image can be significantly inflated compared to the noise in the low‐ and high‐energy images, and the amount of increase is dependent on the material enhancement ratio. We demonstrate this effect Fig. 10 using simulated noise. This consideration points again to the advantage of using energy combination with high material enhancement ratio. In Eq. 1, a second term, RMSE, was included due to the observation that in some situations, the measured data deviated from a linear trend [e.g., Fig. 5(c)]. In such cases, using the SD alone would likely result in an underestimation of the uncertainty in the measurement; the RMSE would more fully capture the residual deviation between the measurements and the calibration curve, and therefore result in a more conservative estimation of the LLOQ. Furthermore, we chose to use the RMS of the SD at all eight ICs, instead of using the SD calculated at individual IC levels.^21^ This was because the estimation of the SD from mere five repetitions would result in too large uncertainty, and including all eight ICs helped greatly reduce the uncertainty. This choice was considered to be justified as the CT number spanned a relatively small range across the IC range investigated, and the difference between the SDs at the lowest and highest ICs is expected to be small (<4%).

A major limitation of the present study is that the same phantoms were used for calibration and measurement, potentially introducing a favorable bias. To assess the magnitude of this effect, we applied the calibration data obtained from the medium phantom to the other two phantoms. At 80/140Sn kVp (Fig. 6A), this resulted in a slight overestimation of IC in the small phantom (by 0.2–0.3 mg/ml for IC <=1 mg/ml, and 10‐20% for IC >=2 mg/ml) and a more significant underestimation of IC in the large phantom. The latter was mainly due to the large shift in CT numbers at 80 kVp in the large phantom [Fig. 4(a)]. A reduced size dependency was observed for 100/140Sn kVp [Fig. 6(b)].

For patient imaging, additional sources of uncertainty may include CT number nonuniformity, scanner drift, and patient motion, which could introduce additional uncertainties to the measured CT number that were not accounted for in the present study. To assess the potential impact of CT number inaccuracies, we tried artificially shifting the CT number in the low‐ or high‐energy image by 3 HU (Fig. 7). Such a shift was sufficient to cause an error of ~1 mg/ml in the predicted IC. On the other hand, if the CT number is shifted in both the low‐ and high‐energy images by a similar amount, the predicted IC is minimally affected.

Thus, in order to achieve reliable iron quantification, it is paramount to ensure accurate calibration of the CT number, the iron enhancement ratio, and the conversion factor. One potential approach to address these needs would be to always scan a calibration phantom with the patient. The calibration phantom should contain reference standard solutions spanning an adequate IC range. Preferably, phantoms of multiple sizes should be available to match the patient size, which would help address the size dependency observed in this study. Another limitation of the present study is that it was limited to one scanner and one scan session. Interscanner and intersession variability will likely further increase the uncertainty in the measured CT number and therefore worsen the LLOQ. These effects could also be mitigated if simultaneous calibration scans are to be performed.

While there are multiple factors in a patient study that may worsen the LLOQ compared to a phantom study, one potentially favorable factor is the possibility of using a large volume‐of‐interest (VOI) in patient images, which could help reduce noise and improve LLOQ. In the present study, the VOI was limited by the vial size (VOI = 3.1 cm^3^, spanning four slices). For comparison, a previous human study^13^ used an average VOI of 19.4 cm^3^ for the liver. The iron storage in liver is relatively uniform,^24^ and therefore the use of a large VOI could be justified. The improvement in LLOQ due to volume averaging is demonstrated in Fig. 8. The relationship could be fitted to a power function with an index of ~‐0.3. Note that this represents a less rapid change than what would be expected from quantum noise alone (−0.5), likely due to the presence of additional sources of uncertainties, such as CT number nonuniformity.

In summary, we report in this work the LLOQ of iron quantification using DSCT and its dependence on patient size, dose level, energy combination, and reconstruction method. Iron quantification at clinically important levels was achieved in a small and a medium phantom, but proved difficult in a large phantom. The chief challenges of achieving lower LLOQ include producing wider spectral separation and ensuring accurate calibration. DECT could potentially complement MRI‐based methods for iron quantification in patients with severe iron overload.

## CONFLICT OF INTEREST

The authors have no other relevant conflicts of interest to disclose.
